# A prospective study of immune responses in patients with lung metastases treated with stereotactic body radiotherapy with or without concurrent systemic treatment

**DOI:** 10.21203/rs.3.rs-3547043/v1

**Published:** 2023-11-14

**Authors:** Dan Duda, Eleni Gkika, Elke Firat, Sonja Adebahr, Erika Graf, Alexandra Eichhorst, Gianluca Radicioni, Simon Lo, Simon Spohn, Ursula Nestle, Nils Nicolay, Gabriele Niedermann, Anca-Ligia Grosu

**Affiliations:** Massachusetts General Hospital; Department of Radiation Oncology, University Medical Center Freiburg, Faculty of Medicine, University of Freiburg, Germany; Department of Radiation Oncology, University Medical Center Freiburg, Faculty of Medicine, University of Freiburg, Germany; Department of Radiation Oncology, University Medical Center Freiburg, Faculty of Medicine, University of Freiburg, Germany; Institute of Medical Biometry and Statistics, Medical Center, Faculty of Medicine, University of Freiburg, Germany; Department of Radiation Oncology, University Medical Center Freiburg, Faculty of Medicine, University of Freiburg, Germany; Department of Radiation Oncology, University Medical Center Freiburg, Faculty of Medicine, University of Freiburg, Germany; Department of Radiation Oncology, University of Washington School of Medicine, Seattle, USA; Department of Radiation Oncology, Medical Center - University of Freiburg, Freiburg, Germany; Department of Radiation Oncology, Medical Center, Faculty of Medicine, University of Freiburg, Germany; Department of Radiation Oncology, University Medical Center Freiburg, Faculty of Medicine, University of Freiburg, Germany; Department of Radiation Oncology, Faculty of Medicine, University of Freiburg, 79106 Freiburg; Medical Center - University of Freiburg, Freiburg, Germany

**Keywords:** SBRT, immunotherapy, immune response, lung metastases, systemic treatment, dose and fractionation

## Abstract

We prospectively evaluated the effects of stereotactic body radiotherapy (SBRT) on circulating immune cells. Patients with oligo-metastatic and oligo-progressive pulmonary lesions were treated with SBRT with (cSBRT) or without (SBRT group) concurrent systemic treatment (chemotherapy or immune checkpoint blockade) using different fractionation regimes. Immunoprofiling of peripheral blood cells was performed at baseline, during, at the end of SBRT, and at the first and second follow-ups. The study accrued 100 patients (80 with evaluable samples). The proportion of proliferating CD8+ T-cells significantly increased after treatment. This increase remained significant at follow-up in the SBRT group, but not in the cSBRT group and was not detected with doses of >10Gy per fraction indicating that lower doses are necessary to increase proliferating T-cells’ frequency. We detected no favorable impact of concurrent systemic treatment on systemic immune responses. The optimal timing of systemic treatment may be post-SBRT to leverage the immune-modulating effects of SBRT.

## Introduction

Stereotactic body radiotherapy (SBRT) is emerging as an essential treatment modality in the oligometastatic setting^1–5^. SBRT can provide local tumor control through significant cytotoxic effects, due to the induction of the double-strand breaks, but can also have systemic effects on the anti-tumor immune response, as previously described^6,7^. Several studies have suggested that using SBRT with sub-ablative doses can lead to immunogenic cell death in the irradiated lesion, and induce immune-mediated abscopal effects outside the irradiation field^8,9^. These effects remain poorly understood, and it is unclear whether the combination of SBRT with systemic treatments including immune checkpoint blockade (ICB) can lead to greater systemic responses because of potential antagonistic interactions. The question of optimal dose and fractionation and timing of immunotherapy or chemotherapy for the enhancement of immune modulation remains of great importance.

We have recently reported that SBRT can induce a significant decrease in the mean absolute counts of CD8 + cytotoxic T lymphocytes (CTLs) and CD4 + T-cells in early-stage non-small cell lung cancer (NSCLC) patients^10^. Nevertheless, the proportion of proliferating CD4 + and CD8 + T-cells among peripheral blood lymphocytes significantly increased after SBRT. Additionally, we have shown that the significant increases in the proportion of CD8 + and CD4 + proliferating T-cells compared to pre-treatment values were only detected in the NSCLC patients who received SBRT with 10Gy or less per fraction.

In this study, we evaluated the effects of SBRT on circulating immune cells when using different fractionation regimes, with or without systemic treatment (including ICB) in the oligometastatic and oligoprogressive disease settings in the lung.

## Materials and Methods

### Patients and study design.

The prospective study was conducted in the Departments of Radiation Oncology of the University Medical Center Freiburg, Germany, and the Massachusetts General Hospital (MGH) and Harvard Medical School, Boston, USA, according to the Declaration of Helsinki. The study was registered in the German trials registry (DRKS 00011266). All patients gave written informed consent according to institutional and federal guidelines. The institutional ethics committees approved the study protocol (LAPIS trial, EK 38/16, Freiburg and MGH IRB Agreement #:2016D009860).

The prospective study included of two arms, depending on the localization of the tumors (lung or liver). The study enrolled patients with primary tumors or oligometastatic/oligoprogressive disease treated with SBRT with or without concurrent systemic treatments such as chemotherapy or immunotherapy (Supplementary Table 2). Each subgroup was analyzed separately, per protocol. We have previously reported the results in early-stage NSCLC^10^. Herein we present the results of the patients with metastatic pulmonary lesions treated with SBRT or hypofractionated radiotherapy with or without systemic treatment. We used immunoprofiling of peripheral blood cells by longitudinal assessment at first SBRT fraction (baseline), during and at the end of SBRT, and at first (FU1) and second (FU2) follow-up (1½ and 4½ months after SBRT, respectively).

### Treatment planning and treatment delivery.

Patients were immobilized in a supine position with a customized vacuum cushion system and received a 4D/CT or a 4D/PET-CT. Patients with peripheral tumors not abutting the chest wall received 3 × 18.75Gy to the D50% such that 95% of the PTV received a minimum of 45 Gy (3 × 15Gy, 80% of the nominal dose) and a dose maximum between 110 and 120%^11^. Depending on the proximity to the central bronchial system and for tumors abutting or overlapping with the chest wall, a total dose of 50Gy in 5 fractions of 10Gy^12^ or 60Gy in 8 fractions of 7.5Gy for central tumors^13^ or 66 Gy in 12 fractions for ultra-central tumors was applied, as previously described^10^. One patient was treated with hypofractionated palliative radiotherapy with 3Gy per fraction up to a total dose of 39 Gy(major deviation). The dose prescription was chosen so that 95% of the PTV received at least the nominal fraction dose, and 99% of the PTV received a minimum of 90% of the nominal dose. The dose maximum within the PTV was chosen to be more than 110% but less than 120% of the prescribed dose. Response to treatment was assessed at the same time points according to the Response Evaluation Criteria in Solid Tumors (RECIST) using thoracic CT and 18F-FDG PET/CT, the latter being mandatory in case of suspected disease progression.

### Flow cytometric analysis.

Blood samples were collected before treatment (baseline), 1 day after (during), at the end (end), at the 1st follow-up (FU1: six weeks after the end of SBRT), and the 2nd follow-up (FU2: 3 months after FU1). Peripheral blood mononuclear cells (PBMCs) were isolated and frozen until use. Not all samples were available from each patient at each time point. The reason for the missing samples was that there were either not collected or had insufficient cells for all analyses.

Frozen PBMCs were thawed, washed, and resuspended in RPMI 1640 media. Cells were then counted, and live/death staining was done using Zombie Red Fixable Viability stain (BioLegend). Cell surface markers were stained with a mixture of antibodies (1:200 dilution each) for 20 minutes at 4°C. To detect nuclear antigens, samples were fixed and permeabilized using the FoxP3 Fixation/Permeabilisation kit from eBioscience. To assess cytokines, cells were incubated in RPMI 1640 media (10^6^ cells/ml) with PMA (50 ng/ml), Ionomycin (1μg/ml), and BFA (1:1000) for 5h. A live/death staining was then applied followed by cell surface marker staining. Cells were fixed with IC Fixation buffer (eBioscience) and stained for intracellular markers for 30 minutes at room temperature. Cells were stained in 4 different multicolor panels: T cell proliferation and exhaustion markers: CD3-FITC, CD4-BV510, CD8-APC, PD-1-PE-Cy7 (EH12.1), Tim3-PE (F38–2E2), CTLA-4-BV605 (BNI3), CD45RA-PerCP-Cy5.5 (HI100), CCR7-AF700 (G043H7), Ki67-BV421 (Ki-67); cytokines: CD3-FITC (OKT3), CD4-BV510 (OKT4), CD8-APC (HIT8a), IFNγ^−^BV421 (B27), IL-17A-PE (BL168); Treg and activation markers: CD3-FITC, CD8-APC, CD4-BV510, CD25-PE-Cy7 (BC96), CD127-PE (A019D5), ICOS-PerCP-Cy5.5 (C398.4), FoxP3-BV421 (206D); MDSCs: HLA-DR-AF700 (L243), CD11b-PE (ICRF44), CD33-APC (P67.6). All antibodies were purchased from BioLegend except for the anti-PD-1-PE-Cy7 antibody, obtained from BD Biosciences. Samples were analyzed on a Cytoflex S flow cytometer (Beckman Coulter). The online-only information shows the gating strategy (Supplementary Figs. 1 and 2).

### Statistical analysis.

The null hypothesis for the primary endpoint was that the probability p of an increase in CD8 + counts at FU1 compared to baseline was p ≤ 50%, to be tested against the alternative that p > 50%. With 50 patients per subgroup, an exact one-sided binomial test at a 5% significance level had at least 80% power to reject the null hypothesis if p > 68.5% applied, and the null hypothesis would be rejected if at least 32 out of 50 patients experienced an increase (exact significance level 3.25%, STPLAN version 4.5). Intra-individual changes in blood biomarkers compared to baseline were examined using a mixed effects model for repeated measures, and multiple comparisons were corrected using the Benjamini, Krieger, and Yekutiely method to control a False Discovery Rate of 5% within variables over time. In a second step, we determined the standardised changes from baseline. Secondary endpoints included changes in other T-cell subsets at all time-points.

Overall (OS) and progression-free survival (PFS) were calculated from the start of SBRT and estimated according to the Kaplan–Meier method. For event-free patients, the observations for OS were censored at the date of the last contact and for PFS at the time of the last imaging. No patients died between baseline and end of treatment. To investigate the correlation between parameters significantly changed at FU1 or FU2 compared to baseline, PFS was calculated from FU1 or FU2 in landmark analyses in the patients still at risk (alive and without progression at FU1 and FU2), respectively.

All p-values except for the primary null hypothesis were two-sided and considered significant if below 5%. The statistical analysis used Prism (Prism V.8, GraphPad Software) and SPSS software (IBM, SPSS, v27).

## Results

### Patient characteristics.

Between 2016–2022, the study accrued 63 patients with oligoprogressive or oligometastatic disease and received no concurrent systemic treatment within 3 months before SBRT (SBRT group) of whom 50 patients with 55 lesions were evaluable by flow cytometry (6 did not receive radiotherapy, 7 patients did not have sufficient blood cells). The median time between the last treatment and SBRT was 25 months. Additionally, a second group which consisted of 37 patients who received concurrent systemic treatment (12 immunotherapy, 18 chemotherapy, or other systemic treatments) in combination with SBRT (cSBRT group) was included in the study. Of the 37 patients, 30 patients with 32 lesions were evaluable in this group (5 patients received no radiotherapy and 2 withdrew consent), of whom 12 received concurrent immunotherapy and 18 concurrent chemotherapy or other systemic treatments.

A total of 25 patients in the SBRT group and 18 patients in the cSBRT group had oligoprogressive disease (OPD) showing signs of progression in one metastatic lesion while having further measurable metastases without signs of progression that were not treated with other local treatments. Patients and treatment characteristics are shown in Table 1 and Supplementary Table 1.

### Treatment outcomes.

With a median follow-up of 30 months, the median OS was 30 months in the SBRT group and 53 months for cSBRT group, and the median PFS was 13 months in the SBRT group and 5 months for cSBRT group. Of note, patients with concurrent ICB treatments had a median PFS of 24 months. In the SBRT group, only one patient developed local progression and declined further treatments, 18 patients developed a regional progression treated with systemic therapy, radiotherapy (mostly SBRT), or resection, and 6 patients developed distant metastases. One patient developed a loco-regional recurrence, 1 patient developed a local recurrence in combination with distant metastases, and 1 patient developed a regional progression in combination with this metastasis. In the cSBRT group, 11 patients developed a regional progression, 7 patients developed distant metastases, and 1 patient had local and distant progression. All types of progression and subsequent treatments are summarized in Supplementary Table 2.

In the 25 patients in the SBRT group with oligoprogressive disease, 15 showed progression during follow-up while 9 had stable disease (one refused CT scans at follow-up). In the cSBRT group, of the 18 patients with oligoprogressive disease; 5 developed a progression, and 18 metastases were stable or showed a partial remission (Supplementary Table 3).

In exploratory analyses, we detected no correlation between PFS and the biological effective dose (BED) (SBRT group: HR per Gy = 0.995, 95%CI: 0.978–1.012, p = 0.54, cSBRT group: HR per Gy = 1.000, 95%CI: 0.975–1.026, p = 0.98) or the dose per fraction (HR = 0.995, 95%CI: 0.920–1.075, p = 0.89, cSBRT: HR = 0.994, 95%CI: 0.846–1,168, p = 0.94) in either of the groups. Furthermore, there was no difference in the PFS between patients with centrally located tumours vs peripheral tumours in both groups respectively (p = 0.87, p = 0.23).

### Circulating lymphocyte kinetics.

An increase in the absolute counts of circulating CD8 + CTLs at FU1 compared to baseline (the primary study endpoint) was detected only in 12% of the patients in the SBRT and 20% of the patients in the cSBRT group.

### Association between circulating lymphocyte changes and PFS.

In landmark analyses, the median PFS from the first follow-up in the SBRT group was 19 months for the patients without an increase in the CD8 + CTLs and was not reached in the patients with an increase in the CD8 + CTLs (HR = 0.484, 95% CI 0.060–3.927, p = 0.49). In the cSBRT group, the median PFS from the first follow-up was 6 vs 3 months in patients without vs with an increase in the CD8 + CTLs (HR 0.944, 95% CI 0.233–3.818 p = 0.93).

### Changes in lymphocyte subsets.

**Figure 1:**
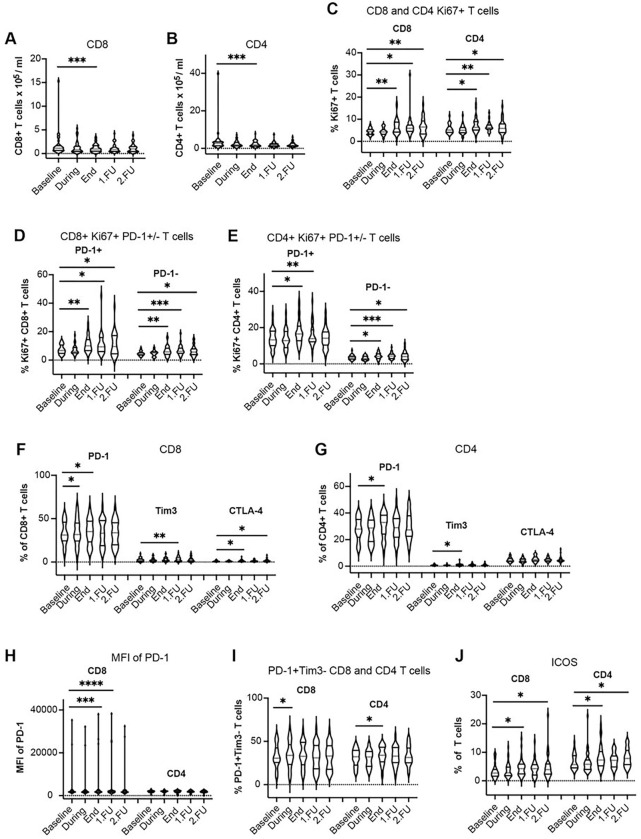
SBRT induces transient lymphodepletion and increased proliferation of CD8^+^ and CD4^+^ circulating T-cells in oligometastatic patients without systemic treatment. **a-b** Absolute counts of circulating CD8^+^ **a** and CD4^+^ T-cells **b**. **c** Fractions of Ki-67^+^CD8^+^ and Ki-67^+^CD4^+^ T-cells, **d** Fractions of Ki67^+^PD-1^+^/ Ki67^+^PD-1^−^ CD8^+^ T-cells and **e** Ki-67^+^PD-1^+^ / Ki-67^+^PD-1^−^ CD4^+^ T-cells. **f** Fraction of PD-1^+^, Tim3^+^ and CTLA-4^+^ CD8^+^ T-cells and **g** PD-1^+^, Tim3^+^ and CTLA-4^+^ CD4^+^ T-cells. **h** Median fluorescence intensity (MFI) of PD-1 expression on CD8^+^ and CD4^+^ T-cells. **i** Fraction of activated PD-1^+^Tim3^−^ CD8^+^ and CD4^+^ T-cells. **j** Fraction of ICOS^+^ CD8^+^ and CD4^+^ T-cells. * p<0.05, ** p<0.01, *** p<0.001, **** p<0.0001 from mixed effects model for repeated measures with Geisser-Greenhouse correction and Benjamini, Krieger, and Yekutieli for the false Discovery Rate, two-sided. Data are shown as median values (center lines, red) and interquartile ranges.

There was a decrease in the mean absolute counts of CD8 + CTLs and CD4 + T-cells compared to pre-treatment values, which was more prominent at the end of treatment in the SBRT group compared to the cSBRT group (Figs. 1a–b, 3a–b, Table 2, Supplementary Fig. 3a-b Supplementary Table 4). This finding is consistent with the changes seen after SBRT in patients with early-stage primary lung tumors^10^. Furthermore, the standardized change from baseline was greater in the group with concurrent systemic treatment (cSBRT group) compared to the SBRT group until the first follow-up (Supplementary Table 4).

In the SBRT group, the proportion of proliferating CD4 + and CD8 + T-cells among peripheral blood lymphocytes (CD3 + cells) increased significantly at the end of treatment and remained significant at FU1 and FU2 (Figs. 1c, 3c, Supplementary Fig. 3c, Table 2). These increases occurred in the PD-1 + and PD-1−subsets of CD4 + and CD8 + T-cells (Fig. 1d–e, 3d–e, Supplementary Fig. 3d-e, Table 2).

In contrast, the fractions of proliferating CD8 + and CD4 + T cells T-cells were significantly increased only at the end of treatment in patients who received SBRT with systemic therapy (Fig. 3c–e). The increase in the fraction of proliferating T-cells was delayed and transient (at FU1 only) in the cSBRT group patients who received ICB and increased at the end of treatment only in CD8 + T-cells in patients who received additional chemotherapy (Fig. 3k–l, Table 2).

Most importantly, the effect size of standardized changes from baseline in proliferating T-cells was large in the SBRT group but only moderate to large in the cSBRT group and considerably lower compared to the SBRT group (Supplementary Table 4). In the cSBRT group, there was no difference in the standardized changes from baseline in proliferating T-cells between patients receiving ICB and patients receiving other systemic treatments at the end of treatment compared to baseline (CD8 + T-cells 0.89 vs 0.90, CD4 + T-cells 0.49 vs 0.45, respectively).

### Changes in circulating lymphocyte phenotypes after SBRT.

**Figure 2:**
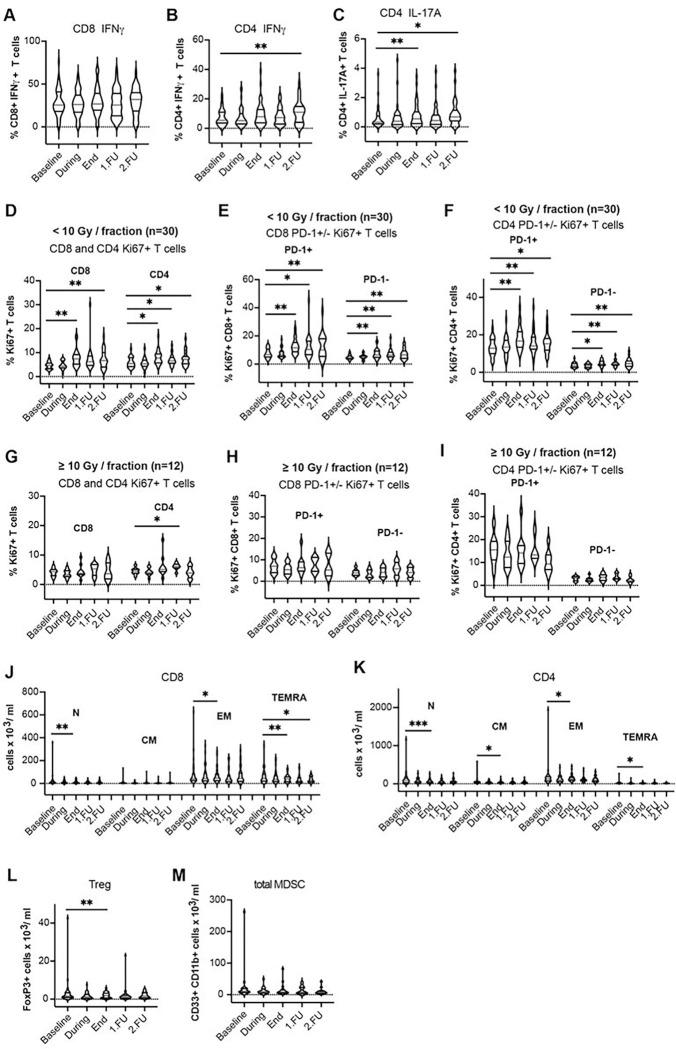
Cytokine expression and SBRT dose-dependent effects on CD8^+^ and CD4^+^ circulating T-cell proliferation post-treatment in oligometastatic patients without systemic treatment. Expression of IFN-γ in CD8^+^ T-cells **a,** CD4^+^ T-cells **b** and expression of IL-17A in CD4^+^ T-cells **c**, upon re-stimulation. **d-i** Fraction of Ki-67^+^CD8^+^ and Ki-67^+^CD4^+^ T-cells after SBRT using doses of <10 Gy per fraction **d-f** versus doses ³10Gy per fraction **g-i**. **j** Absolute cell counts for naïve and memory CD8^+^ T-cell subpopulations and **k** naïve and memory CD4^+^ T-cell subpopulations. **l** Absolute Treg counts. **m** Absolute MDSC counts. * p<0.05, ** p<0.01, *** p<0.001, **** p<0.0001 from mixed effects model for repeated measures with Geisser-Greenhouse correction and Benjamini, Krieger, and Yekutieli for the false Discovery Rate, two-sided. Data are shown as median values (center lines, red) and interquartile ranges.

**Figure 3:**
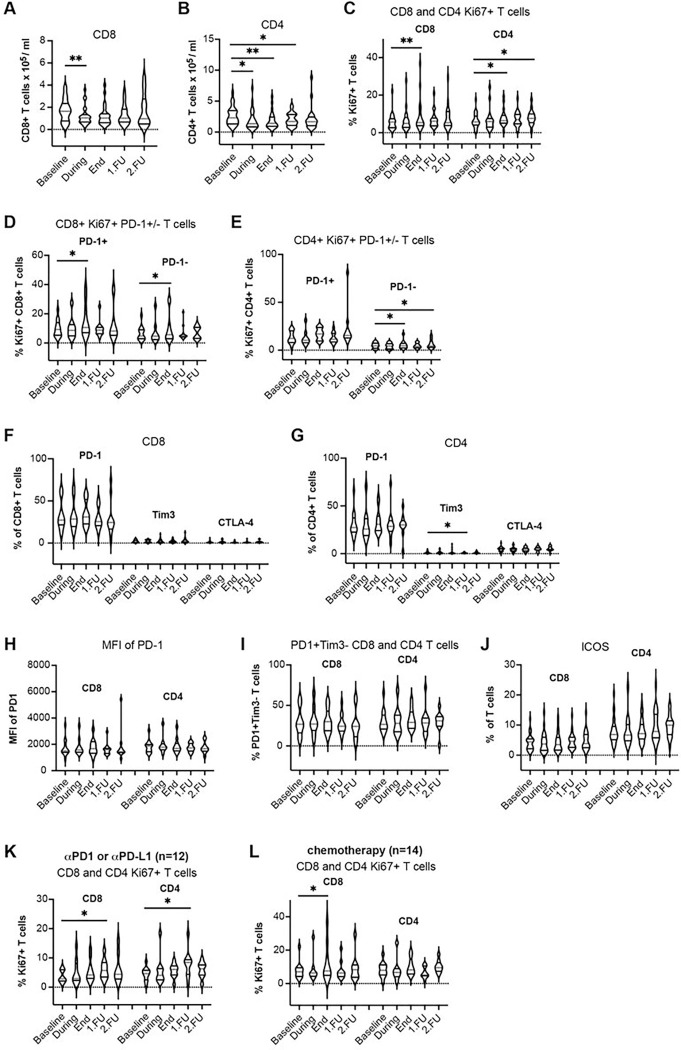
Transient lymphodepletion after SBRT and increased proliferation of CD8^+^ and CD4^+^ circulating T-cells in oligometastatic patients with systemic treatment. **a-b** Absolute counts of CD8^+^ **a** and CD4^+^ circulating T-cells **b**. **c-e** Fractions of Ki-67^+^CD8^+^ and Ki-67^+^CD4^+^ T-cells **c**, Ki67^+^PD-1^+^/ Ki67^+^PD-1^−^ CD8^+^ T-cells **d**, and Ki-67^+^PD-1^+^ / Ki-67^+^PD-1^−^ CD4^+^ T-cells **e**. **f** Fraction of PD-1^+^, Tim3^+^ and CTLA-4^+^ CD8^+^ T-cells. **g** Fraction of PD-1^+^, Tim3^+^ and CTLA-4^+^ CD4^+^ T-cells. **h** Median fluorescence intensity (MFI) of PD-1 expression on CD8^+^ and CD4^+^ T-cells. **i** Fraction of activated PD-1^+^Tim3^−^ CD8^+^ and CD4^+^ T-cells. **j** Fraction of ICOS^+^ CD8^+^ and CD4^+^ T-cells. **k** Fraction of Ki67^+^CD8^+^ and Ki67^+^CD4^+^ T-cells after SBRT plus ICB. **l** Fraction of Ki67^+^CD8^+^ and Ki67^+^CD4^+^ T-cells after SBRT plus chemotherapy. * p<0.05, ** p<0.01, *** p<0.001, **** p<0.0001 from mixed effects model for repeated measures with Geisser-Greenhouse correction and Benjamini, Krieger, and Yekutieli for the false Discovery Rate, two-sided. Data are shown as median values (center lines, red) and interquartile ranges.

**Figure 4:**
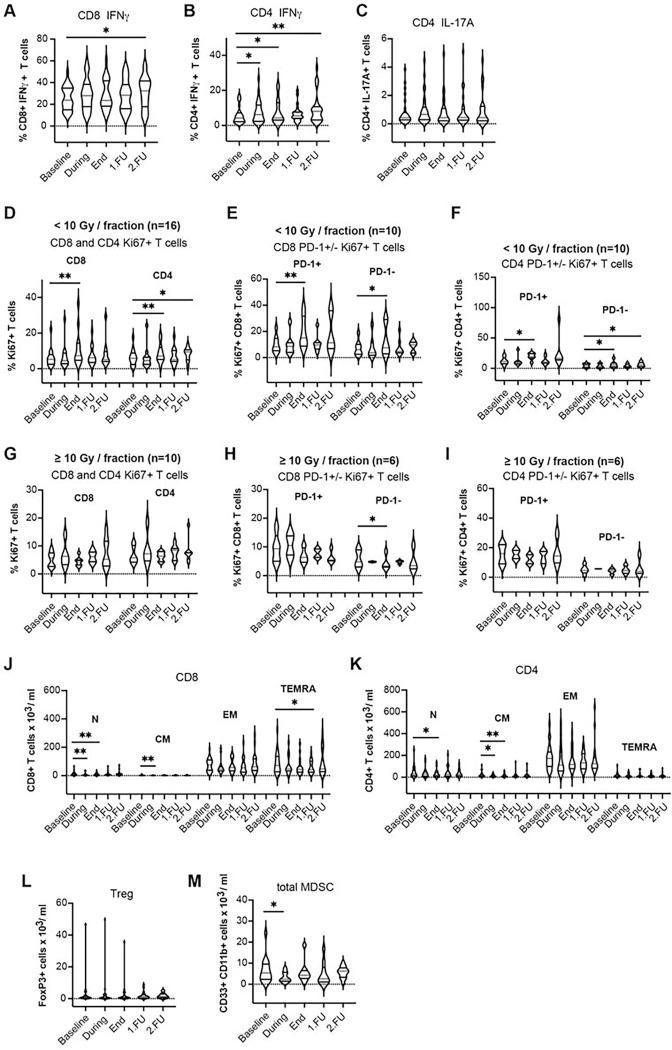
Expression of cytokines and SBRT dose-dependent effects on CD8^+^ and CD4^+^ circulating T-cell proliferation post-treatment in oligometastatic patients with systemic treatment. Expression of IFN-γ in CD8^+^ T-cells **a** and CD4^+^ T-cells **b** and expression of IL-17A in CD4^+^ T-cells **c**, upon re-stimulation. **d-i** Fraction of Ki-67^+^CD8^+^ and Ki-67^+^CD4^+^ T-cells after SBRT using doses of <10 Gy per fraction **d-f** versus doses ³10Gy per fraction **g-i**. **j** Absolute cell counts for naïve and memory CD8^+^ T-cell subpopulations and **k** naïve and memory CD4^+^ T-cell subpopulations. **l** Absolute Treg counts. **m** Absolute MDSC counts. * p<0.05, ** p<0.01, *** p<0.001, **** p<0.0001 from mixed effects model for repeated measures with Geisser-Greenhouse correction and Benjamini, Krieger, and Yekutieli for the false Discovery Rate, two-sided. Data are shown as median values (center lines, red) and interquartile ranges.

Median fluorescence intensity (MFI) of PD-1 immunostaining was also higher at the end of treatment and at FU1 for the CD8 + T-cells, indicative of an increased expression level which was significant in the SBRT group (Figs. 1h, 3h, Supplementary Fig. 3h, Table 2) but the effect size based on the standardized change from baseline was small (Supplementary Table 4). Additionally, the fractions of T-cells expressing the activation marker IFN-γ increased from baseline through to FU2 and the change was significant at the end of treatment in the cSBRT group, with a small effect size based on the standardized change from baseline (Supplementary Table 4). The fractions of T-cells expressing the activation marker IL-17A increased at the end of treatment (CD4 + T-cells) and FU2 (CD4 + T-cells) in the SBRT group with a small effect size (Figs. 2a–c, 4a–c, Supplementary Fig. 4a-c, Table 2, Supplementary Table 4). Overall, there was a decrease in naïve and memory CD8 + and CD4 + T-cell subpopulations after SBRT and at FU (Figs. 2j–k, 4j–k, Supplementary Fig. 4d-e, Table 2, Supplementary Table 4).

The fractions of CD8 + and CD4 + T-cells expressing inducible costimulatory (ICOS) significantly increased at the end of treatment and FU2 in the SBRT group (Fig. 1j, Table 2, Supplementary Fig. 3j), while the effect size based on the standardized change from baseline was small (Supplementary Table 4). In the cSBRT group, the increase of ICOS expression at FU was greater than in the SBRT group, but the change did not reach statistical significance (Fig. 3j, Table 2, Supplementary Fig. 3j, Supplementary Table 4).

Circulating Treg fractions decreased from baseline through to FU2, and the change was significant at the end of treatment in the SBRT group (Figs. 2l, 4l, Supplementary Fig. 4f, Table 2). The effect size based on the standardized change from baseline was small (Supplementary Table 4).

Circulating MDSC fraction significantly changed only during SBRT in the patients with systemic treatment (cSBRT group) (Figs. 2m, 4m, Supplementary Fig. 4g). The effect size based on the standardized change from baseline was moderate (Supplementary Table 4).

In the cSBRT group, the expression of PD-1, TIM3, and CTLA-4 was detected only on a minority of circulating T-cells, indicating that most circulating T-cells were not terminally exhausted. In contrast, in the SBRT group, the circulating T-cells appeared more exhausted (Figs. 1f–g, 3f–g, Table 2, Supplementary Fig. 3f-g, Supplementary Table 4).

The fraction of activated PD-1 + Tim3− T-cells increased during (for CD8 + T-cells) and at the end of SBRT (for CD4 + T cells) in the SBRT group (Figs. 1i, 3i, Supplementary Fig. 3i). In both cases the effect size based on the standardized change from baseline was small (Supplementary Table 4). The kinetics of all immune cell subsets are summarized in Table 2 and the standardized changes from baseline in Supplementary Table 4.

### Effect of dose per fraction on lymphocyte changes.

In addition, we performed further sub-group analyses after stratifying for RT dose per fraction, using 10Gy as the cut-off point based on our previous findings in early-stage NSCLC patients^10^. Patients treated with less than 10Gy showed a significant increase in the proportion of CD8 + and CD4 + proliferating T-cells compared to pre-treatment values at the end of treatment and follow-up. This effect was not seen in patients treated with 10Gy or more. The increases were significant for both SBRT and cSBRT groups at the end of treatment while only in the SBRT group this effect was maintained at follow-ups (Figs. 2d–i, 4d–i).

## Discussion

To the best of our knowledge, this is the largest study evaluating the systemic immune modulatory effects of SBRT with or without concurrent systemic treatment, including immunotherapy, in lung metastatic disease. We found promising PFS rates in all groups, with the longest in the cSBRT /ICB group.

The primary endpoint of our study was not met, as only 12% of the patients in the SBRT and 20% of the patients in the cSBRT group experienced an increase in the absolute counts of CD8 + T-cells at FU1 compared to baseline. Furthermore, our findings confirm the transient lymphopenia effects of SBRT in lung metastatic disease, as previously reported for SBRT in early-stage NSCLC patients^10^. This effect was more pronounced in patients who received concurrent systemic treatment. Importantly, we also validated our previous finding that SBRT-induced lymphopenia is followed by an increased T-cell proliferation, which may include tumor-specific T-cells^14–17^, both with or without systemic treatment. Although the effects were generally similar in both groups, the effect size of standardized changes from baseline was considerably higher in the group without concurrent systemic treatment. Interestingly, the increased T-cell proliferation was maintained at the first and second follow-ups in the patients without systemic treatment (SBRT group), suggesting that the timing of systemic treatment is critical for maximizing the immune-related effects of SBRT. Importantly, concurrent systemic treatment did not lead to an augmentation of this systemic immunomodulatory effect. Previous studies in mice have shown that the therapeutic effect of SBRT combined with anti-PD1 was considerably reduced when immunotherapy was initiated before irradiation^18^.

Additionally, in patients with oligo progressive disease, without concurrent systemic treatment (SBRT group), T-cells seemed to be more exhausted at baseline than in those with systemic treatment. In patients with oligometastatic disease, SBRT has the potential to both reduce tumor burden and promote T-cell responses against micrometastases^19,20^. We did not observe any abscopal effects, in patients with untreated but stable metastases. Furthermore, the median PFS was longer in the SBRT group than that in the cSBRT group, but this result is confounded by the different tumor histologies, as most solid tumors are highly heterogeneous and evolve dynamically during treatment^21^. Interestingly, PFS was significantly longer in patients with concurrent ICB treatment. However, we did not find any additional impact of ICB or chemotherapy on the systemic immune responses at the time-points evaluated in this study.

As previously described, the increased proliferation of CD8^+^ and CD4^+^ circulating T-cells occurred only in the patients treated with 10 Gy or less per fraction^10^. This effect could be attributed to the upregulation of three-prime repair exonuclease 1 (TREX1)^6,22^. These results were validated for SBRT in the metastatic disease setting.

Our study has several limitations. Due to the different duration of SBRT regimens, post-treatment evaluations were not time matched. Furthermore, we included patients with different histologies, which carry a differential prognosis. Given the heterogeneity in tumor histologies, these SBRT outcomes need to be validated in a disease-specific manner. Nevertheless, the parameters of radiation dose and fractionation scheme, administration schedule, and target volume are all likely to have a crucial influence on the ability of radiotherapy to elicit immunostimulatory effects that can be exploited with immunotherapies (notably ICIs) towards superior clinical efficacy^22^. This study addressed some of these important open questions.

In conclusion, our study shows that ablative SBRT leads to transient lymphodepletion but a significant increase in the fraction of proliferating CD4^+^ and CD8^+^ circulating T-cells after treatment in lung metastatic disease, consistent with our findings in primary lung cancer. The increase was present at the end of treatment and follow-up but was more pronounced in the SBRT group than in the cSBRT group, and only when using doses of 10Gy or less per fraction. These data might help future studies of optimal integration of ICBs with SBRT in oligo-metastatic or oligo-progressive disease, for example starting after the end of SBRT with doses of less than 10Gy per fraction.

## References

[1] Gomez D. R. Local Consolidative Therapy Vs. Maintenance Therapy or Observation for Patients With Oligometastatic Non-Small-Cell Lung Cancer: Long-Term Results of a Multi-Institutional, Phase II, Randomized Study. Journal of clinical oncology : official journal of the American Society of Clinical Oncology 37, 1558–1565, doi:10.1200/jco.19.00201 (2019). 31067138 PMC6599408

[2] Iyengar P. Consolidative Radiotherapy for Limited Metastatic Non-Small-Cell Lung Cancer: A Phase 2 Randomized Clinical Trial. JAMA Oncol 4, e173501, doi:10.1001/jamaoncol.2017.3501 (2018). 28973074 PMC5833648

[3] Palma D. A. Stereotactic Ablative Radiotherapy for the Comprehensive Treatment of Oligometastatic Cancers: Long-Term Results of the SABR-COMET Phase II Randomized Trial. Journal of clinical oncology : official journal of the American Society of Clinical Oncology 38, 2830–2838, doi:10.1200/jco.20.00818 (2020). 32484754 PMC7460150

[4] Siva S. Single-Fraction vs Multifraction Stereotactic Ablative Body Radiotherapy for Pulmonary Oligometastases (SAFRON II): The Trans Tasman Radiation Oncology Group 13.01 Phase 2 Randomized Clinical Trial. JAMA Oncol 7, 1476–1485, doi:10.1001/jamaoncol.2021.2939 (2021). 34455431 PMC8404145

[5] Chalkidou A. Stereotactic ablative body radiotherapy in patients with oligometastatic cancers: a prospective, registry-based, single-arm, observational, evaluation study. Lancet Oncol 22, 98–106, doi:10.1016/s1470-2045(20)30537-4 (2021). 33387498

[6] Demaria S. Radiation dose and fraction in immunotherapy: one-size regimen does not fit all settings, so how does one choose? J Immunother Cancer 9, doi:10.1136/jitc-2020-002038 (2021). PMC8031689 33827904

[7] Formenti S. C. & Demaria S. Systemic effects of local radiotherapy. Lancet Oncol 10, 718–726, doi:10.1016/s1470-2045(09)70082-8 (2009). 19573801 PMC2782943

[8] Theelen W. Randomized phase II study of pembrolizumab after stereotactic body radiotherapy (SBRT) versus pembrolizumab alone in patients with advanced non-small cell lung cancer: The PEMBRO-RT study. 36, 9023–9023, doi:10.1200/JCO.2018.36.15_suppl.9023 (2018).

[9] Theelen W. Pembrolizumab with or without radiotherapy for metastatic non-small-cell lung cancer: a pooled analysis of two randomised trials. Lancet Respir Med 9, 467–475, doi:10.1016/s2213-2600(20)30391-x (2021). 33096027

[10] Gkika E. Systemic immune modulation by stereotactic radiotherapy in early-stage lung cancer. NPJ Precis Oncol 7, 24, doi:10.1038/s41698-023-00358-z (2023). 36864234 PMC9981559

[11] Guckenberger M. Definition of stereotactic body radiotherapy: principles and practice for the treatment of stage I non-small cell lung cancer. Strahlentherapie und Onkologie : Organ der Deutschen Rontgengesellschaft ... [et al] 190, 26–33, doi:10.1007/s00066-013-0450-y (2014). PMC3889283 24052011

[12] Bezjak A. Safety and Efficacy of a Five-Fraction Stereotactic Body Radiotherapy Schedule for Centrally Located Non-Small-Cell Lung Cancer: NRG Oncology/RTOG 0813 Trial. Journal of clinical oncology : official journal of the American Society of Clinical Oncology 37, 1316–1325, doi:10.1200/jco.18.00622 (2019). 30943123 PMC6524984

[13] Adebahr S. LungTech, an EORTC Phase II trial of stereotactic body radiotherapy for centrally located lung tumours: a clinical perspective. The British journal of radiology 88, 20150036–20150036, doi:10.1259/bjr.20150036 (2015). 25873481 PMC4628529

[14] Min B. Spontaneous T Cell Proliferation: A Physiologic Process to Create and Maintain Homeostatic Balance and Diversity of the Immune System. Front Immunol 9, 547, doi:10.3389/fimmu.2018.00547 (2018). 29616038 PMC5868360

[15] Kim C. G. Dynamic changes in circulating PD-1(+)CD8(+) T lymphocytes for predicting treatment response to PD-1 blockade in patients with non-small-cell lung cancer. European journal of cancer 143, 113–126, doi:10.1016/j.ejca.2020.10.028 (2021). 33302114

[16] Gros A. Prospective identification of neoantigen-specific lymphocytes in the peripheral blood of melanoma patients. Nat Med 22, 433–438, doi:10.1038/nm.4051 (2016). 26901407 PMC7446107

[17] Gros A. Recognition of human gastrointestinal cancer neoantigens by circulating PD-1+ lymphocytes. J Clin Invest 129, 4992–5004, doi:10.1172/jci127967 (2019). 31609250 PMC6819109

[18] Wei J. Sequence of αPD-1 relative to local tumor irradiation determines the induction of abscopal antitumor immune responses. Science immunology 6, doi:10.1126/sciimmunol.abg0117 (2021). 33837124

[19] Huang A. C. T-cell invigoration to tumour burden ratio associated with anti-PD-1 response.Nature 545, 60–65, doi:10.1038/nature22079 (2017). 28397821 PMC5554367

[20] Lin A. J., Roach M., Bradley J. & Robinson C. Combining stereotactic body radiation therapy with immunotherapy: current data and future directions. Transl Lung Cancer Res 8, 107–115, doi:10.21037/tlcr.2018.08.16 (2019). 30788240 PMC6351396

[21] Vitale I., Shema E., Loi S. & Galluzzi L. Intratumoral heterogeneity in cancer progression and response to immunotherapy. Nat Med 27, 212–224, doi:10.1038/s41591-021-01233-9 (2021). 33574607

[22] Galluzzi L., Aryankalayil M. J., Coleman C. N. & Formenti S. C. Emerging evidence for adapting radiotherapy to immunotherapy. Nature reviews. Clinical oncology, doi:10.1038/s41571-023-00782-x (2023). 37280366

